# EnzyBase: a novel database for enzybiotic studies

**DOI:** 10.1186/1471-2180-12-54

**Published:** 2012-04-11

**Authors:** Hongyu Wu, Hairong Lu, Jinjiang Huang, Guodong Li, Qingshan Huang

**Affiliations:** 1 State Key Laboratory of Genetic Engineering, School of Life Sciences, Fudan University, Shanghai 200433, China; 2 Shanghai High-Tech United Bio-Technological R&D Co, Ltd, Shanghai 201206, China

## Abstract

**Background:**

Enzybiotics are becoming increasingly recognized as potential alternative therapies for drug-resistant bacteria. Although only a few enzybiotics are currently well characterized, much information is still missing or is unavailable for researchers. The construction of an enzybiotics database would therefore increase efficiency and convenience in investigating these bioactive proteins and thus help reduce or delay the recent increase in antibiotic resistance.

**Description:**

In the present manuscript, we describe the development of a novel and original database called EnzyBase, which contains 1144 enzybiotics from 216 natural sources. To ensure data quality, we limited the source of information to authoritative public databases and published scientific literature. The interface of EnzyBase is easy to use and allows users to rapidly retrieve data according to their desired search criteria and blast the database for homologous sequences. We also describe examples of database-aided enzybiotics discovery and design.

**Conclusion:**

EnzyBase serves as a unique tool for enzybiotic studies. It has several potential applications, e.g. *in silico *enzybiotic combination as cocktails, and novel enzybiotic design, in response to continuously emerging drug-resistant pathogens. This database is a valuable platform for researchers who are interested in enzybiotic studies. EnzyBase is available online at http://biotechlab.fudan.edu.cn/database/EnzyBase/home.php.

## Background

Antibiotic abuse is, in part, responsible for the dramatic increase in the resistance of pathogens to traditional antibiotics [1]. Superbugs, such as MRSA and NDM-1, frequently and seriously threaten public safety [2,3]. Consequently, the need to develop new classes of antibiotics with novel mechanisms of action against drug-resistant pathogens is becoming very urgent. Enzybiotics [4-8] and antimicrobial peptides (AMPs)[9] have attracted much attention as potential substitutes for conventional antibiotics.

In the present manuscript, enzybiotics are referred to as bacterial cell wall-degrading enzymes, including lysins, bacteriocins, autolysins, and lysozymes. The most important characteristics of enzybiotics are their novel mechanisms of antibacterial action and capacity to kill antibiotic-resistant bacteria [10]. Another significant feature of certain enzybiotics is their low probability of developing bacterial resistance [11]. Compared with AMPs, enzybiotics are large, heat-labile, and narrow-spectrum types of antimicrobial proteins. Consequently, enzybiotics are not always suitable antimicrobial agents. Despite this, certain enzybiotics have been well characterized and widely used. Lysostaphin [12-15] and lysozymes [16-18] are the most studied enzybiotics in regards to their clinical or food applications. Furthermore, despite their apparent limitations in medicine, their potency against multi-drug-resistant pathogens should not be ignored. Therefore, an enzybiotic specific database that not only mobilizes research on enzybiotics, but also makes it more efficient and convenient, needs to be constructed.

Over the past decade, many databases have been developed for AMPs. These databases, including APD [19,20], ANTIMIC [21], CAMP [22], BACTIBASE [23,24], PhytAMP [25], PenBase [26], Defensins [27], CyBase [28], and peptaibols Peptaibol [29], contain AMP sequences from diverse origins or specific families and accordingly have accelerated and stimulated research on AMPs. Conversely, the majority of the sequenced enzybiotics are stored in the manually annotated UniProt/Swiss-Prot [30] database or scattered in the scientific literature. As a result, it is difficult to find information on enzybiotics for recent users. Developing a central database that stores information on enzybiotics is warranted by investigators to promote their research on enzybiotics discovery and design.

The idea of constructing a database that stores information on enzybiotics arose from our own research experience. We found that we constantly had to query information on enzybiotics from public databases, such as UniProt, and scientific literature. Thus, we decided to construct a database that simplified our research efforts, and comprehensively collected this information. EnzyBase, a novel and original database for enzybiotics studies, was developed and currently contains 1144 enzybiotics from 216 natural sources. This database provides a platform for current users to comprehensively and conveniently research enzybiotics and can be useful for exploring and designing novel enzybiotics for medical use.

### Construction and content

EnzyBase was built on an Apache HTTP Server (V2.2.14) with PHP (V5.2.13) and MySQL Server (V5.1.40) as the back-end, and Personal Home Page (PHP), HyperText Markup Language (HTML) and Cascading Style Sheets (CSS) as the front-end. Apache, MySQL, and PHP were preferred as they are open-source software and platform independent, respectively, making them suitable for academic use. The web server and all parts of the database are hosted at Information Office of Fudan University, Shanghai, China.

All enzybiotic sequences were collected manually from the annotated UniProt/Swiss-Prot database or scientific literature. Each enzybiotic without the UniProt link had been excluded. The enzybiotics collected in EnzyBase database are primarily from natural sources, with the exception of genetically-modified sequences. Additional physicochemical data of each enzybiotic was either calculated via Bioperl programs or identified from scientific literature via a PubMed search. All of the collected information was classified and filled into six relational tables in MySQL. For each enzybiotic, a unique identification number (i.e., enzy id) was assigned, beginning with the prefix EN. Each entry also contains general data, such as protein name, protein full name, producer organism, simple function annotation and protein sequence, domains, 3D structure, and relevant references. For all proteins that already exist in the UniProt, Interpro [31], and/or PDB [32] databases, hyperlinks to these databases were created in EnzyBase. Additional physicochemical data, including calculated isoelectric point (pI) and charge at pI, are also provided. Moreover, minimal inhibitory concentrations (MICs) are included, if data are available. The BlastP program (BLASTP V2.2.25+) [33,34] was used for sequence homology searches against EnzyBase.

### Utility and discussion

#### Database description

**Figure 1:**
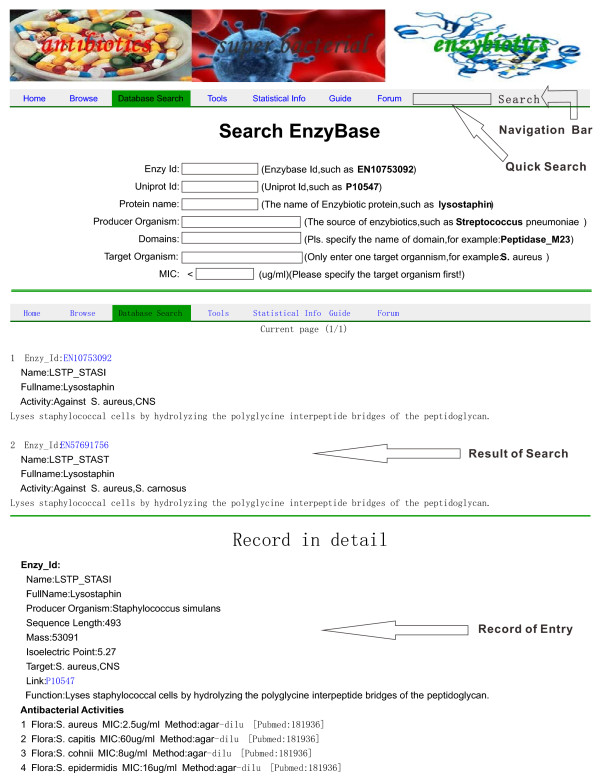
**Screen shots of the EnzyBase search interface**. Screen shots of the EnzyBase search interface showing the advanced search and result views. Please note that not all fields are shown.

EnzyBase supplies a user-friendly web interface, so that users can easily query and retrieve information on enzybiotics. A concise navigational interface that contains the database browse, search, tools, statistical information, and guide, as well as a forum, were designed to generate a clearly structured database layout that enables fast and easy navigation (Figure 1).

As a web-based database, all data can be accessed and retrieved directly from the web browser. The database browse interface provides the users with a function of navigating the entire database, whereas the search interface provides the users with the function of retrieving their desired information using either the "quick" or "advanced" options. A "quick" search can be performed using only keywords, while the "advanced" search offers the possibility to specify seven separate fields, namely enzy id, uniprotKB entry number (i.e., uniprot id), protein name, producer organism, domains, target organism, and MIC value. The user can query the database by either one condition (excluding MIC, which requires the type of target organism to be initially stated) or a combination of various conditions. Every enzybiotic has its own results page that contains comprehensive information, including general information, antibacterial activities, sequence, structures, domains, and references. The general information consists of enzy id, protein name, protein full name, producer organism, protein mass, calculated pI, antibacterial activity, and simple function annotations. EnzyBase also provides hyperlinks to other databases, such as UniProt, InterPro, PDB, and PubMed, which allows for easier navigation within the World Wide Web pertaining to additional information on enzybiotics. The tools interface permits the use of BLASTP against EnzyBase, which enables users to search the database for homologous sequences, and then copy obtained results for subsequent research. Owing to limitations of disk space on the host site, we did not implement a local BLASTP against the NCBI database but instead supplied a hyperlink to the BLASTP on the NCBI website. The statistical info interface provides data on sources for enzybiotics, the distribution of sequence length, protein mass, calculated protein pI, and domains (please refer to the 'Statistical description and findings' section below for more information). The guide interface provides simple instructions for potential users on how to use the functions of EnzyBase. Additionally, the forum tools, which are based on UseBB, a free forum software, have been integrated into the database to provide information on updates, bug reports, and user discussions.

### Statistical description and findings

**Figure 2:**
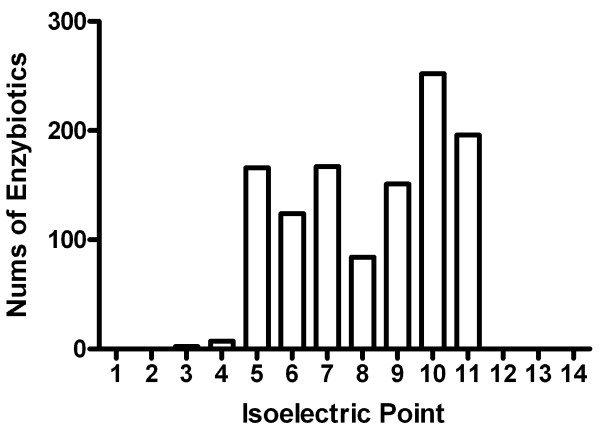
**Distribution of calculated isoelectric points for the enzybiotics within EnzyBase**. Every bar indicates the number of enzybiotics calculated to have their isoelectric point range from pI 1 to 14.

The current version of EnzyBase possesses 1144 enzybiotics from 216 natural sources. The length of the enzybiotic sequences range from 72 to 2337 amino acids. Table 1 presents the top 10 sources for enzybiotics in EnzyBase. The majority (99.2%) of enzybiotics have a calculated pI ranging from 4 to 11 (Figure 2).

**Table 1:** Top 10 sources of enzybiotics in EnzyBase

Rank	Producer organisms	Numbers of enzybiotics
1	***Staphylococcus aureus***	142

2	***Enterococcus faecalis***	136

3	***Bacillus cereus***	73

4	***Streptococcus pneumoniae***	66

5	***Bacillus thuringiensis***	57

6	***Staphylococcus phage***	55

7	***Listeria monocytogenes***	28

8	***Staphylococcus epidermidis***	27

9	***Clostridium perfringens***	21

10	***Enterococcus faecium***	21

All enzybiotics in EnzyBase contain 55 domains, and only 24 enzybiotics have known 3D structures. The top 10 domains for the enzybiotics within EnzyBase are presented in Table 2. The Amidase_domain is the top domain (till 2012-2-6). In fact, this domain is carried by 392 enzybiotics, representing ca. 34% of the total number of enzybiotics in EnzyBase. Thus, it appears that many of the recorded enzybiotics are amidase like.

**Table 2:** Top 10 domains in EnzyBase

Rank	Interpro Id	Domain Name	Numbers of enzybiotics
1	IPR002502	Amidase_domain	392

2	IPR007921	CHAP	224

3	IPR017853	Glycoside_hydrolase_SF	188

4	IPR002053	Glyco_hydro_25	188

5	IPR013781	Glyco_hydro_subgr_catalytic	187

6	IPR002901	Mano_Glyc_endo_b_GlcNAc	169

7	IPR018392	Peptidoglycan-bd_lysin	147

8	IPR013667	SH3_5_bac	141

9	IPR002482	Peptidoglycan-bd_Lysin_subgr	141

10	IPR003646	SH3-like_bac	134

### Applications

The EnzyBase can be used as a tool to aid researchers in exploring the use of enzybiotics or for designing novel enzybiotics. The most prominent weakness of enzybiotics is their narrow spectrum of antibacterial activity. However, a combination of enzybiotics with different spectra of antibacterial activities and/or different mechanisms of action could be used against a broad spectrum of bacterial infections and/or their resistant strains. Through the use of EnzyBase, users can quickly find a series of enzybiotics with optimum antibacterial activities against specific pathogens, and then combine them as a cocktail to measure their therapeutic effect against bacterial infectious diseases. Similar approaches have been successfully used to design phage cocktail therapies for the treatment of infections [35]. For novel enzybiotics design, users could search for potential domains with high antibacterial activities against specific pathogens on EnzyBase and then combine them to create chimeric enzybiotics. For instance, to search for effective antimicrobial proteins against mastitis-causing pathogens, researchers created a novel chimeric peptidoglycan hydrolase fusion protein between lysostaphin and the endolysin of phage B30, which possesses their respective enzymatic domains, and is capable of degrading both streptococcal and staphylococcal peptidoglycans [36]. Thus, the quantity and quality of the data entered in EnzyBase appears to be very important for successfully applying it in such research applications.

In the future, we plan to implement updates, assess the data quality continuously, and integrate some structural analysis tools, such as RasMol [37], and certain web2.0 functions, such as Wiki, into EnzyBase to improve its interactivity with users and improve research in the field of enzybiotics design and structure function exploration.

## Conclusions

In summary, EnzyBase is a comprehensive and web-accessible database of enzybiotics. The current version of EnzyBase has 1144 entries. The database can be queried either by using simply keywords or by combinatorial conditions searches. EnzyBase may aid in enhancing our current understanding of enzybiotics and their mechanisms of action. Its potential applications include the *in silico *development of combinations of enzybiotics (e.g., cocktails) and the construction of novel enzybiotics against various bacterial infectious diseases. Thus, the database may have implications in the development of new drugs for medical applications.

## Availability and requirements

EnzyBase is freely available for academic users at http://biotechlab.fudan.edu.cn/database/EnzyBase/home.php.

## Authors' contributions

HW developed the web interface, designed the rational database scheme, and qualified the data. HL and JH primarily contributed to inputting the data into the current database, as well as in writing the manuscript. GL and QH conceived of the initial idea of the database, provided direction for its development, and revised the subsequent drafts of this manuscript. All authors read and approved the final manuscript.
